# Approach to vertigo in children: A single-center experience

**DOI:** 10.1097/MD.0000000000047499

**Published:** 2026-01-30

**Authors:** Sevgi Çirakli, Emre Öztürk, Burak Sariaydin

**Affiliations:** a Department of Pediatric Neurology, Ordu University, Ordu, Turkey; b Department of Pediatric, Medicalpark State Hospital, Ordu, Turkey.

**Keywords:** cause, child, treatment, vertigo

## Abstract

Pediatric vertigo is not a rare symptom, and it has a significant impact on the child’s quality of life. The aim was to describe the etiologies, diagnostic steps, and treatment outcomes in pediatric vertigo. Forty patients who presented with vertigo between May 2018 and September 2024 were evaluated. The etiology was determined by physical examinations, family histories, blood tests, and cranial imaging methods of the patients, and treatments were planned accordingly; 22 (55%) of the patients were female, and 18 (45%) were male. The ages of the patients ranged from 3 to 18 (median 14). The diagnosis was psychogenic vertigo in 5 patients (12.5%), vertigo associated with migraine in 3 patients (7.5%), epileptic vertigo in 3 patients (7.5%), vestibuloneurinitis in 1 patient (2.5%), vertigo occurring after chickenpox infection in 2 patients (5%), vertigo due to orthostatic hypotension in 4 patients (10%), iron deficiency anemia in 4 patients (10%), intracranial subdural hemorrhage due to trauma in 1 patient (2.5%), B12 deficiency in 5 patients (12.5%), and benign positional paroxysmal vertigo of childhood in 12 patients (30%). Vertigo is a disturbing symptom in children, and severe cases require immediate medical attention. Although treatment varies depending on the etiology, we recommend cranial imaging to all patients.

## 1. Introduction

Vertigo is an illusion of spinning. It occurs due to unequal neural activity between the right and left vestibular nuclei. Vertigo may develop as a result of sudden unilateral damage to the vestibular end organ, the vestibular nerve or its nucleus, or the vestibulo-cerebellum, which inhibits the ipsilateral vestibular nucleus.^[1]^

Vestibular disorders are an important disorder in children, and dizziness is the most common one. It can cause loss of motor skills, imbalance, restlessness, and significant psychological stress. In very young children, the clinic may present only as restlessness or nystagmus.^[2]^ Vertigo negatively affects the quality of life of patients because it causes balance problems and walking difficulties.^[3]^

Understanding the development and maturation of the vestibular system may provide more information about vestibular test results and the etiology of dizziness in different age groups. Compared with the cochlea, the vestibular organs develop earlier and faster.^[4]^

Vertigo may occur for many different reasons, including benign paroxysmal vertigo, vertebrobasilar insufficiency, vestibular neuritis/labyrinthitis, cerebellar infarction/hemorrhage, Meniere disease, cerebellopontine tumors, traumatic vertigo, multiple sclerosis, otosclerosis, vertiginous migraine, and epilepsy. Causes of vertigo often do not produce vital sign abnormalities. The most common cause in children is benign paroxysmal vertigo of childhood (BPVC), while other types of vertigo are observed less frequently. Our single-center study will contribute to the literature in terms of determining regional causes.

Medical history, accompanying findings, and physical examination are very important to distinguish vertigo, and observation of caregivers is very important in young children. Audiological testing and an ear, nose, and throat consultation are requested if necessary. Cranial imaging is essential in children to rule out emergencies.

## 2. Materials and methods

### 2.1. Case data

Forty patients who applied to our hospital’s pediatric neurology and pediatric emergency clinic due to vertigo between May 2018 and September 2024 were evaluated. Patients’ files were scanned retrospectively. Approval numbered 2024/150 was received from our university ethics committee.

The patient files were reviewed retrospectively, and all patients were evaluated by the same pediatric neurologist. Patients between the ages of 0 and 18 were included in the study. Patients who had sufficient follow-up and agreed to participate in the study were included in the study. Vertigo was evaluated by physical examinations, family histories, blood tests (B12 deficiency ≤ 200 pg/mL and iron deficiency anemia ≤ 10 gr/dL), and cranial imaging methods of the patients, and treatment was planned accordingly. Patients were diagnosed with vertigo based on clinical description and caregiver observation.

### 2.2. Statistical analysis

IBM SPSS (Statistical Package for the Social Sciences) Statistics for Windows, version 21.0, was used for the analysis data (SPSS Inc., Chicago). Descriptive statistical analysis was used. While evaluating the study data, categorical variables were expressed as n (%), normally distributed continuous variables as mean ± standard deviation, and non-normally distributed continuous variables as median and minimum-maximum.

## 3. Results

Twenty-two (55%) of the patients were female and 18 (45%) were male. The ages of the patients ranged from 3 to 18 (median 14). All patients underwent blood tests, examination by the ear, nose, and throat department, electroencephalogram (EEG), and cranial magnetic resonance imaging (MRI).

**Figure 1.**
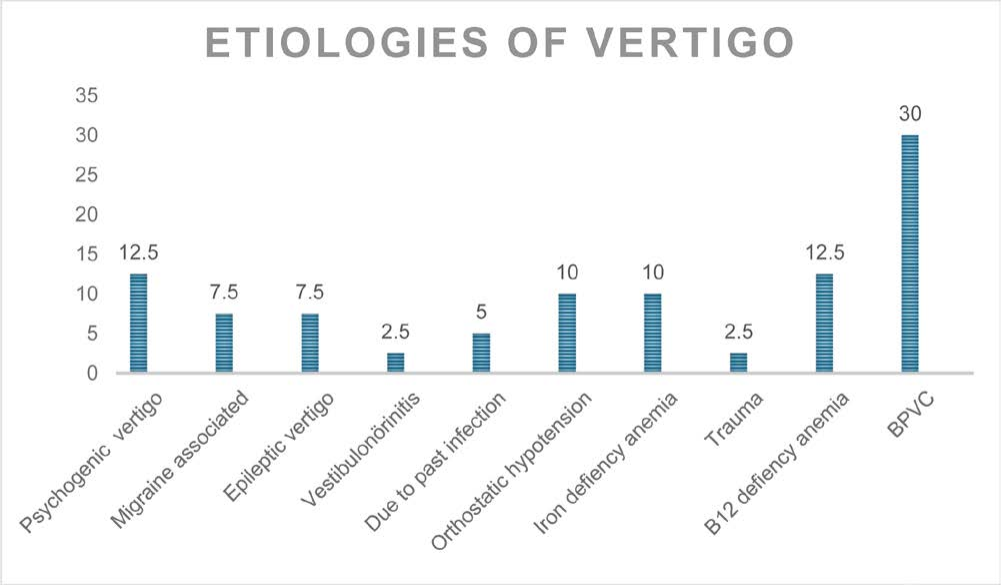
Etiologies of vertigo. BPVC = benign positional paroxysmal vertigo of childhood.

Different etiologies were detected in the patients. Etiologies are high stress levels and no findings in the tests, psychogenic vertigo was diagnosed, 3 patients had vertigo associated with migraine, 3 patients had epileptic vertigo, 1 patient had vestibuloneurinitis, 2 patients had vertigo following chickenpox infection, 4 patients had vertigo due to orthostatic hypotension, 4 patients had iron deficiency anemia, 1 patient had intracranial subdural hemorrhage due to trauma, 5 patients had B12 deficiency, and 12 patients had BPVC. The frequency of etiological causes is summarized in Figure 1.

Cranial MRI was performed on all patients. Subdural hematoma was observed in one of the patients on imaging, and it had shifted intracranial structures, and the patient was taken into emergency surgery.

A 17-year-old male patient who was taken into emergency surgery because of traumatic vertigo. For this reason, we recommend cranial MRI for patients with central nervous system findings. It is very important in a patient with central nervous system pathology such as intracranial mass, arteriovenous malformation, and dizziness.

Of the 5 patients evaluated as having psychogenic vertigo, 3 were female (60%) and 2 were male (40%). All 3 patients evaluated as having migraine-associated vertigo had a family history of migraine. EEG was performed in vertigo cases with no etiological cause detected, and 3 patients were evaluated as having epileptic vertigo due to abnormal EEG. Antiepileptic treatment was initiated. Of the 3 patients with epileptic vertigo, 1 (33.3%) had no family history of epilepsy and 2 (66.6%) had a family history of epilepsy (Table 1).

**Table 1:** Characteristics of patients.

Number	Age	Gender	Etiology	Additional feature
1	3	Female	B12 deficiency	
2	4	Female	B12 deficiency	
3	6	Male	Iron deficiency	
4	6	Male	Postinfection	
5	7	Male	Iron deficiency	
6	7	Female	Postinfection	
7	8	Female	BPVC	
8	8	Female	BPVC	
9	8	Male	Iron deficiency	
10	8	Male	Epilectic	Epileptic EEG findings
11	9	Male	B12 deficiency	
12	9	Female	Epilectic	Epileptic EEG findings
13	10	Female	Iron deficiency	
14	10	Male	Epilectic	Epileptic EEG findings
15	12	Male	BPVC	
16	12	Male	B12 deficiency	
17	12	Female	Psychogenic	
18	12	Female	Psychogenic	
19	12	Male	Vestibuloneuritis	
20	13	Male	Migraine	
21	14	Male	B12 deficiency	
22	14	Male	BPVC	
23	14	Female	Psychogenic	
24	14	Female	Migraine	
25	14	Male	Orthostatic	
26	14	Male	BPVC	
27	15	Female	Orthostatic	
28	15	Female	BPVC	
29	15	Male	BPVC	
30	15	Male	Psychogenic	
31	16	Female	BPVC	
32	16	Male	Psychogenic	
33	16	Female	Orthostatic	
34	17	Female	Orthostatic	
35	17	Female	Migraine	
36	17	Female	BPVC	
37	17	Male	Trauma	Subdural hemorrhage
38	18	Female	BPVC	
39	18	Female	BPVC	
40	18	Female	BPVC	

BPVC = benign positional paroxysmal vertigo of childhood, B12 = cobalamin, EEG = electroencephalogram.

All patients were asked for evaluation by the ear, nose, and throat department, and 13 cases (32.5%) were diagnosed by the ear, nose, and throat department. Twelve of these patients (30%) were evaluated as having BPVC, which is the highest rate among our patients with vertigo. One patient was evaluated as having vestibuloneurinitis and steroid treatment was started. This patient was started on methyl prednisolone at a dose of 1 mg/kg and tapered off within 10 days. This patient’s clinical condition improved significantly after steroid treatment. One month after treatment, she experienced a mild attack, and instead of steroids, she was given piracetam only. The symptoms subsided within 3 days.

Blood tests were performed on all patients, and 5 (12.5%) patients were observed to have B12 deficiency and 4 (10%) patients were observed to have iron deficiency (22.5%). The patients’ complaints improved after vitamin replacement therapy was initiated. This highlights the importance of blood tests as a first step.

In cases where vertigo is considered to be due to orthostatic hypotension, avoiding sudden movements and getting up from bed slowly are recommended for treatment. Two patients thought to have infectious cerebellitis had chickenpox infection 1 week prior, and their vertigo findings regressed without treatment.

## 4. Discussion

Vertigo in children is a symptom that worries families, and in our study, the most common diagnosis (30%) was BPVC. This rate was also the highest (39%) in a study of 100 patients conducted by Batu et al.^[5]^ In the literature, Riina et al^[6]^ and Niemensivu et al^[7]^ who have extensive vertigo research found BPVC to be the highest.

Psychogenic vertigo was observed as the second most common type (12.5%) in our study, while Batu et al^[5]^ reported psychogenic vertigo as the second most common type (21%). However, the rates of BPVC, psychogenic, and epileptic vertigo were lower in our study. We believe this is because there were more diverse etiologies in our study.

Epileptic vertigo is not listed as an etiological factor in some pediatric studies in the literature. We attribute the high rate in our study to our location in a pediatric neurology unit, as this diagnosis is made using EEGs performed in neurology units.

Varicella is a common viral infection in childhood. Although its incidence has decreased significantly since the introduction of routine vaccination programs in our country, it is still prevalent. It is highly contagious to those without immunity. Acute cerebellitis may occur as a complication following varicella infection. In a large-scale study conducted in Italy on 457 children with chickenpox, the rate of acute cerebellitis was found to be (10.5%).^[8]^ Depending on the severity of acute cerebellitis, symptomatic treatment is recommended, with steroids recommended for severe cases. Two patients with acute cerebellitis who were followed up for varicella infection due to vertigo in our study experienced the resolution of their symptoms with symptomatic treatment.

Headaches can often accompany vertigo, and it can sometimes be difficult to distinguish between migraine-related vertigo and psychogenic vertigo. This is because headaches often accompany cases diagnosed as psychogenic vertigo, where no cause can be found.^[9]^ In our study, headaches were also present independently of vertigo in patients diagnosed with psychogenic vertigo.

Vitamin B12 (also known as cobalamin) is a B vitamin that plays an important role in cell metabolism, particularly DNA synthesis, methylation, and mitochondrial metabolism. Its deficiency can lead to neurological and hematological findings.^[10,11]^ Patients with B12 deficiency can sometimes present with neurological problems such as weakness and imbalance. If a simple blood test identifies the cause and the vitamin deficiency is replaced, clinical improvement is observed if the findings are consistent. In our study, 5 patients diagnosed with vertigo due to B12 deficiency were treated with methylcobalamin, the active form of B12, as a sublingual spray, and all patients’ symptoms were significantly relieved (12.5%).

**Figure 2.**
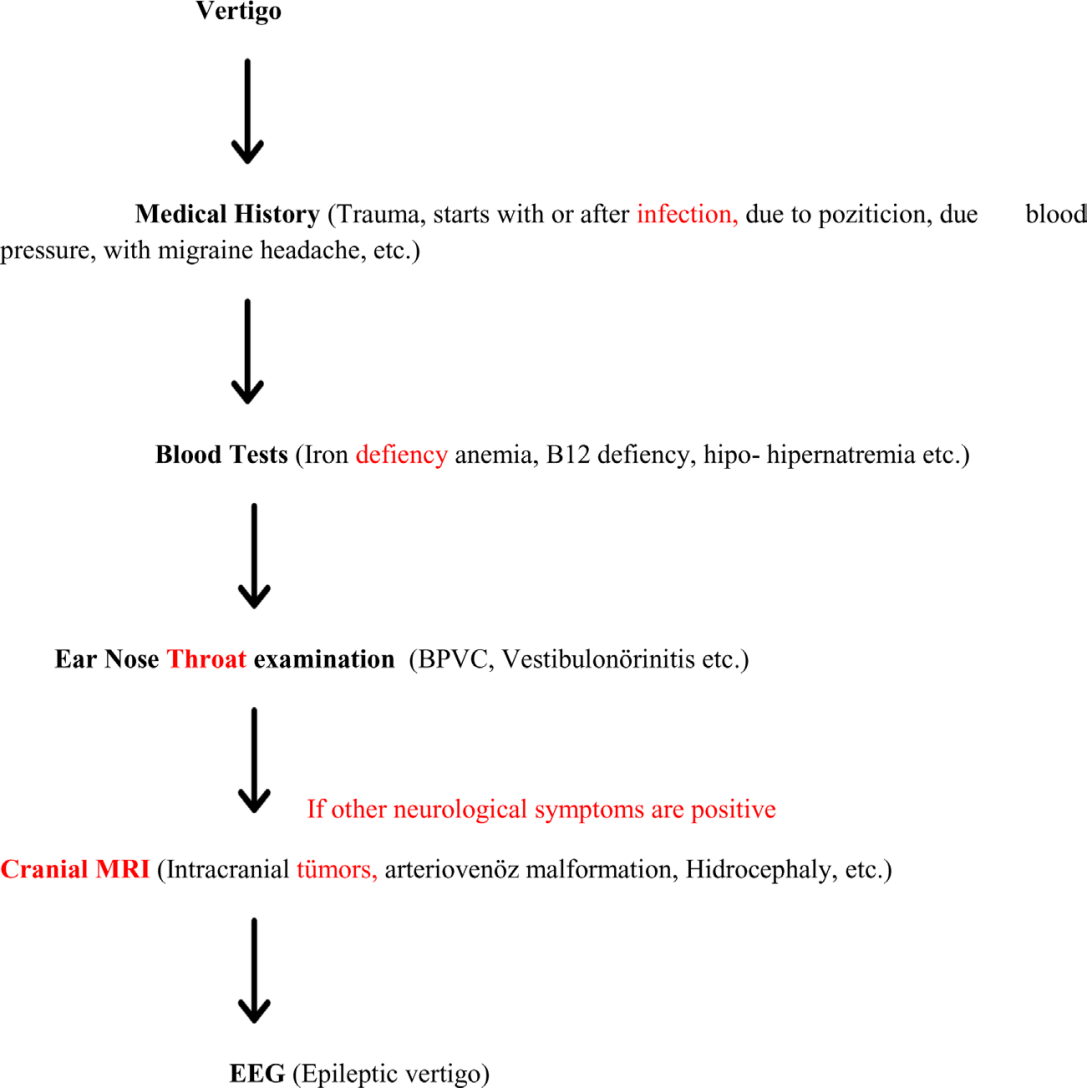
Approach to vertigo in childhood. BPVC = benign positional paroxysmal vertigo of childhood, EEG = electroencephalogram, MRI = cranial magnetic resonance imaging.

Childhood symptoms associated with migraine are classified as recurrence of gastrointestinal disturbance, cyclic vomiting syndrome, abdominal migraine, benign paroxysmal vertigo, and benign paroxysmal torticollis according to ICD-3 criteria.^[12]^ Although the pathogenesis of BPVC is not clearly known, it is thought to be a pathology in the vestibular nucleus and vestibulocerebellar pathways.^[13]^ A correlation has been shown between BPVC and the likelihood of migraine in later ages.^[14]^ Further time is needed to evaluate this correlation in our study. We have created the following algorithm in our clinic to determine the etiology of pediatric patients presenting with vertigo (Fig. 2). We believe that algorithms will be effective and practical in the future when approaching patients in this way.

The retrospective nature of the study, the relatively small number of cases, and the lack of standard diagnostic criteria are the limitations of our study.

## 5. Conclusion

Severe cases of vertigo require immediate emergency medical attention. While treatment varies depending on the etiology. Cranial imaging is useful in selected cases, particularly when neurological findings are present. Vestibular suppressant therapy is administered in cases with normal cranial imaging, and steroid therapy is administered in cases suspected of vestibuloneuritis. Most cases of short-term vertigo do not require treatment, and an EEG is performed to distinguish possible epilepsies presenting with vertigo symptoms. In addition, before performing these tests, we can perform tests for iron deficiency and B12 deficiency, which are common in children and can cause fatigue and vertigo, and provide replacements if necessary. BPVC was the most frequent cause of vertigo in our cohort, followed by psychogenic and epileptic etiologies.

## Acknowledgments

I would like to thank Emre Öztürk and Burak Sariaydin for helping me create this article.

## Author contributions

**Data curation:** Sevgi Çirakli, Emre Öztürk, Burak Sariaydin.

**Formal analysis:** Sevgi Çirakli, Emre Öztürk.

**Investigation:** Sevgi Çirakli.

**Methodology:** Sevgi Çirakli, Burak Sariaydin.

**Writing – original draft:** Sevgi Çirakli, Emre Öztürk, Burak Sariaydin.

## References

[1] Dunker K Schnabel L Grill E Filippopulos FM Huppert D. Recurrent vertigo of childhood: clinical features and prognosis. Front Neurol. 2022;13:1022395. 36247755 10.3389/fneur.2022.1022395 PMC9554238

[2] Kelly EA Janky KL Patterson JN. The dizzy child. Otolaryngol Clin North Am. 2021;54:973–87. 34304898 10.1016/j.otc.2021.06.002

[3] Khan A Liu S Tao F. Current trends in pediatric migraine: clinical insights and therapeutic strategies. Brain Sci. 2025;15:280. 40149800 10.3390/brainsci15030280 PMC11940401

[4] O’Reilly R Grindle C Zwicky EF Morlet T. Development of the vestibular system and balance function: differential diagnosis in the pediatric population. Otolaryngol Clin North Am. 2011;44:251–71, vii. 21474003 10.1016/j.otc.2011.01.001

[5] Batu ED Anlar B Topçu M Turanli G Aysun S. Vertigo in childhood: a retrospective series of 100 children. Eur J Paediatr Neurol. 2015;19:226–32. 25548116 10.1016/j.ejpn.2014.12.009

[6] Riina N Ilmari P Kentala E. Vertigo and imbalance in children: a retrospektive study in Helsinki University otorhinolaryngology clinic. Arch Otolaryngol Head Neck Surg. 2005;131:996–1000. 16301372 10.1001/archotol.131.11.996

[7] Niemensivu R Kentala E Wiener-Vacher S Pyykko I. Evoluation of vertiginous children. Eur Arch Otorhinolaryngol. 2007;264:1129–35. 17503065 10.1007/s00405-007-0329-6

[8] Bozzola E Bozzola M Tozzi AE. Acute cerebellitis in varicella: a ten year caseseries and systematic review of the literatüre. Ital J Pediatr. 2014;40:57. 24942129 10.1186/1824-7288-40-57 PMC4079178

[9] Langhagen T Schroeder AS Rettinger N Borggraefe I Jahn K. Migraine-related vertigo and somatoform vertigo frequently occur in children and rare are often associated. Neuropediatrics. 2013;44:55–8. 23307184 10.1055/s-0032-1333433

[10] Green R Allen LH Bjorke-Monsen A-L. Vitamin B_12_ deficiency. Nat Rev Dis Primers. 2017;3:17040. 28660890 10.1038/nrdp.2017.40

[11] Karatoprak E Sözen G Yilmaz K. How often do neurological disorders lead to dizziness in childhood? Turk Arch Pediatr. 2021;56:249–53. 34104917 10.14744/TurkPediatriArs.2020.43410 PMC8152640

[12] Headache Classification Committee. The international classification of headache disorders, 3rd edition (beta version). Cephalalgia. 2013;33:629–808. 23771276 10.1177/0333102413485658

[13] Winner P. Migraine-related symptoms in childhood. Curr Pain Headache Rep. 2013;17:339. 23961555 10.1007/s11916-013-0339-6

[14] Ralli G Atturo F, de Filippis C. Idiopathic benign paroxysmal vertigo in children, a migraine precursor. Int J Pediatr Otorhinolaryngol. 2009;73:16–8. 10.1016/S0165-5876(09)70004-7 20114149

